# Cancer heterogeneity explored through immune cell deconvolution of the tumor microenvironment

**DOI:** 10.1002/qub2.70037

**Published:** 2026-04-15

**Authors:** Limin Jiang, Chung‐I Li, Rui Xin, Jijun Tang, Yan Guo

**Affiliations:** ^1^ Department of Public Health Sciences University of Miami Miami Florida USA; ^2^ Department of Statistics Cheng Kung University Tainan China; ^3^ Department of Computer Science University of South Carolina Columbia South Carolina USA; ^4^ Faculty of Computer Science and Control Engineering Shenzhen University of Advanced Technology Shenzhen China

**Keywords:** deconvolution, heterogeneity, tumor microenvironment

## Abstract

Cancer is a highly heterogeneous disease, characterized by significant variability across multiple dimensions. This diversity has been extensively studied from various perspectives. In this study, we aim to examine cancer heterogeneity through the lens of immune cell composition within the tumor microenvironment. Transcriptome profiles of 11,274 subjects from 33 cancer types were deconvoluted to infer the composition of 22 immune cell types. A deep learning‐based model was adapted to model a binary outcome for cancer type, tumor versus normal. The Shannon index was used to represent immune cell diversity. Cox proportional hazards regression was used to evaluate the prognostic values of immune cells. Tumor and normal tissues showed significantly different immune cell compositions in 183 of 352 comparisons, with macrophage M0 levels consistently elevated in most tumors except lung adenocarcinoma. ResNet models using immune cell data achieved strong performance in distinguishing tumor versus normal samples (average F1 score = 0.64 when combined with clinical features). Four immune features were significantly associated with disease‐specific survival across three cancer types: macrophage M0 and macrophage M2 in bladder urothelial carcinoma, macrophage M0 in kidney renal clear cell carcinoma, and the Treg/CD8^+^ T cell ratio in skin cutaneous melanoma. Although immune cell diversity varied across cancers, it was not broadly predictive of prognosis, highlighting the cancer type‐specific nature of immune remodeling. Our results demonstrated cancer heterogeneity through the analysis of immune cell composition and diversity using deep learning models and prognostic analysis. The significant findings revealed varying patterns across different cancer types, indicating that no unifying standard can be established across all cancers.

**Abbreviations**
ACC: adrenocortical carcinoma
BLCA: bladder urothelial carcinoma
BRCA: breast invasive carcinoma
CESC: cervical squamous cell carcinoma and endocervical adenocarcinoma
CHOL: cholangiocarcinoma
COAD: colon adenocarcinoma
DLBC: lymphoid neoplasm diffuse large B‐cell lymphoma
ESCA: esophageal carcinoma
GBM: glioblastoma multiforme
HNSC: head and neck squamous cell carcinoma
KICH: kidney chromophobe
KIRC: kidney renal clear cell carcinoma
KIRP: kidney renal papillary cell carcinoma
LAML: acute myeloid leukemia
LGG: brain lower grade glioma
LIHC: liver hepatocellular carcinoma
LUAD: lung adenocarcinoma
LUSC: lung squamous cell carcinoma
MESO: mesothelioma
OV: ovarian serous cystadenocarcinoma
PAAD: pancreatic adenocarcinoma
PCPG: pheochromocytoma and paraganglioma
PRAD: prostate adenocarcinoma
READ: rectum adenocarcinoma
SARC: sarcoma
SKCM: skin cutaneous melanoma
STAD: stomach adenocarcinoma
TGCT: testicular germ cell tumors
THCA: thyroid carcinoma
THYM: thymoma
UCEC: uterine corpus endometrial carcinoma
UCS: uterine carcinosarcoma
UVM: uveal melanoma

## 1 INTRODUCTION

Immune cells play a crucial role in the body’s defense against cancer. Specifically, T cells and natural killer (NK) cells continuously monitor the body for abnormal cells, enabling them to identify and destroy cancer cells at an early stage, thus preventing tumor development [1]. Cytotoxic T lymphocytes (CTLs) recognize cancer‐specific antigens presented on the surface of tumor cells and induce apoptosis [2]. Upon activation against cancer cells, the immune system can develop memory cells that provide long‐term protection. Immune cells can augment the effectiveness of cancer treatments [3]. For instance, immunotherapies such as checkpoint inhibitors rely on reactivating the patient’s immune cells to target and destroy cancer cells. Other treatments, such as vaccines and adoptive cell transfer, directly utilize immune cells. Moreover, immune cells can influence the tumor microenvironment (TME), making it less conducive to cancer growth [4]. They can secrete cytokines that inhibit tumor proliferation and promote an antitumor immune response. By targeting and eliminating circulating tumor cells and micrometastases, immune cells can prevent the spread of cancer to other parts of the body [5]. Furthermore, chronic inflammation, which can promote cancer development, can be modulated by immune cells, particularly regulatory T cells (Tregs) and macrophages, thereby reducing the risk of cancer progression [6]. Immune cells can also shape the evolution of cancer cells through a process known as immunoediting, which includes the elimination of highly immunogenic cancer cells and the establishment of a state of equilibrium in which the immune system controls tumor growth [7].

Recent large‐scale studies have demonstrated that the immune composition of the TME varies significantly across cancer types and is a key determinant of patient prognosis and response to therapy. For example, a pan‐cancer analysis of the Cancer Genome Atlas (TCGA) data revealed that the abundance and type of infiltrating immune cells, particularly cytotoxic T cells and macrophages, are strongly associated with survival outcomes, often surpassing traditional clinical features in prognostic value [8]. Similarly, the development of computational tools such as quanTIseq has enabled the quantification of immune cell fractions from bulk RNA‐seq data, uncovering that immune infiltration is more closely linked to chemokine signaling than to tumor mutational burden and that kinase inhibitors can modulate the immune contexture to enhance immunotherapy efficacy [9].

Single‐cell RNA sequencing has further refined our understanding of immune heterogeneity within tumors. In melanoma, specific subsets of CD8^+^ T cells expressing exhaustion markers and C‐X‐C motif chemokine ligand 13 were found to be enriched in patients responding to PD‐1 blockade, suggesting that the presence of these cells may serve as predictive biomarkers for immunotherapy success [10]. In a comprehensive immunogenomic analysis of over 10,000 tumors across 33 cancer types, six distinct immune subtypes were identified, each associated with unique immune signatures, prognoses, and potential responses to immunotherapy. This study revealed that immune landscape classification can transcend tissue origin and provide a framework for understanding tumor–immune interactions across cancers [11]. Immune cells significantly influence cancer patient survival through various mechanisms involving both direct and indirect interactions with tumor cells and the TME throughout the course of treatment. Immune checkpoint therapies work by blocking inhibitory pathways that suppress T cell activity, thereby enhancing the immune response against tumors [12]. In adoptive cell transfer therapy, immune cells are collected from the patient, activated and expanded ex vivo, and then reintroduced to the patient to fight the cancer. The success of immunotherapies is closely tied to the patient’s immune landscape [13]. The presence and ratio of certain immune cells within the tumor (e.g., high CD8^+^ T cell infiltration, low Treg/CD8^+^ T cell ratio) have been shown to be predictive of patient outcomes. These immune profiles can help stratify patients into different prognostic groups [14, 15].

Given the critical role of immune cells, various techniques have been developed to directly measure their abundance, such as flow cytometry, immunohistochemistry, and mass cytometry. To further advance the study of immune cells, an indirect method, CIBERSORTx [16], was developed to infer immune cell content from RNA‐seq data. CIBERSORTx deconvolutes bulk tissue gene expression data to estimate the proportions of different cell types and, in high‐resolution mode, infers cell type‐specific gene expression profiles. In this study, we utilized CIBERSORTx to comprehensively deconvolute gene expression profiles of 14 cancer types and analyze their immune cell patterns in relation to colinearity, prognosis, and cancer type specificity.

## 2 RESULTS

### 2.1 Pan‐cancer immune cell type distribution

The overview of the data processing is shown in Figure 1. The complete immune cell type dataset used in this study is available in Table S1. Deconvolution of RNA‐seq data for 33 cancer types was performed using CIBERSORTx [16] with the 22 immune cell type signatures (LM22). The distributions of these 22 immune cell types for 14 cancer types, each with more than 20 normal samples, are shown in Figure 2. Cancer types with fewer than 20 normal samples were removed in this analysis. In tumor samples, the most abundant immune cell type, on average, is T cells CD4 memory resting (average proportion: 0.148, min: 0.102, max: 0.220), whereas the least abundant is T cell CD4 naive (average: 2.40 × 10^−4^, min: <10^−9^, max: 9.05 × 10^−4^). In normal samples, the most abundant immune cell type, on average, is T cell CD4 memory resting (average: 0.200, min: 0.125, max: 0.254), and the least abundant is T cell CD4 naive (average: 2.37 × 10^−4^, min: < 10^−9^, max: 2.41 × 10^−4^).

**FIGURE 1:**
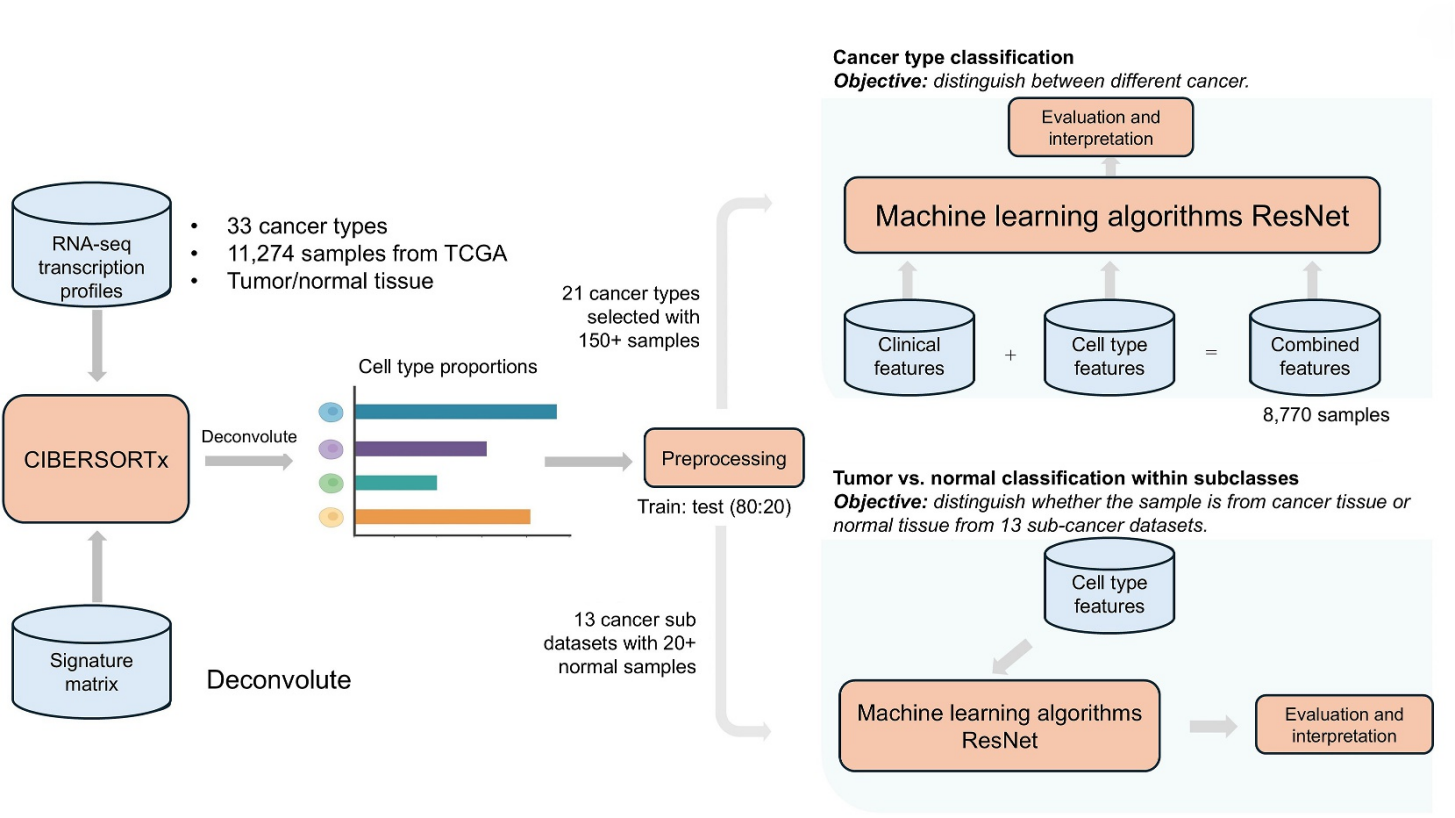
Overview of the study design and workflow.

**FIGURE 2:**
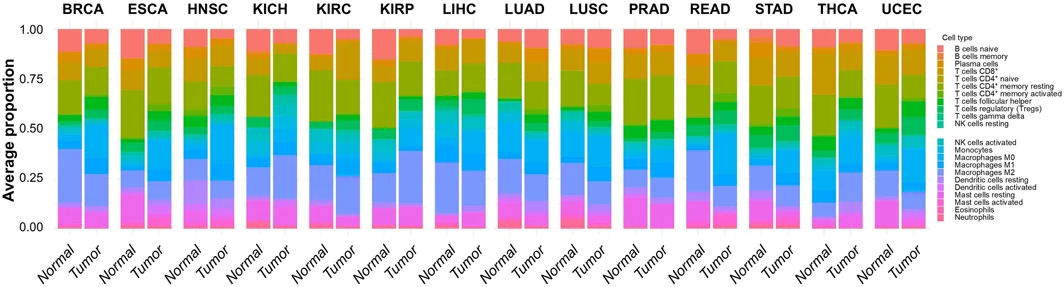
Immune cell composition distribution for cancers with normal samples.

Using stomach cancer as an example, Figure 3 presents a heatmap illustrating immune cell type abundances at single‐sample resolution. The bar plot on the left side of the figure displays the −log_10_‐transformed *p*‐values from *t*‐tests comparing immune cell proportions between tumor and normal samples, highlighting the statistical significance of the observed differences. Colinearities between immune cell types were computed using Pearson’s correlation after aggregating all tumor samples. The correlation coefficients for tumor and normal samples are plotted in Figure 4. The upper right triangle contains correlations computed from normal samples, and the lower left triangle contains correlations computed from tumor samples. For normal samples, there were 2 positive correlations with coefficients > 0.3 (B cells naive–plasma cells [*r* = 0.37], T cell CD8^+^–T cell follicular helper [*r* = 0.36]) and 3 negative correlations with coefficients < −0.3 (T cell CD8^+^–macrophage M2 [*r* = −0.4], T cell follicular helper–macrophage M2 [*r* = −0.34], mast cells resting–mast cells activated [*r* = −0.32]). Age is not associated with any immune cell type. For tumor samples, there were 4 positive correlations with coefficients > 0.3 (plasma cells–B cells naive [*r* = 0.45], T cell CD4 memory activated–T cell CD8^+^ [*r* = 0.35], T cell follicular helper–T cell CD8^+^ [*r* = 0.35], eosinophils–monocytes [*r* = 0.33]) and 3 negative correlations with coefficients < −0.3 (T cell CD4 memory resting–T cell CD8^+^ [*r* = −0.33], macrophage M0–monocytes [*r* = −0.31], macrophage M2–T cell follicular helper [*r* = −0.31]). Age is not associated with any immune cell type. The complete results of the correlation analysis are available in Table S2.

**FIGURE 3:**
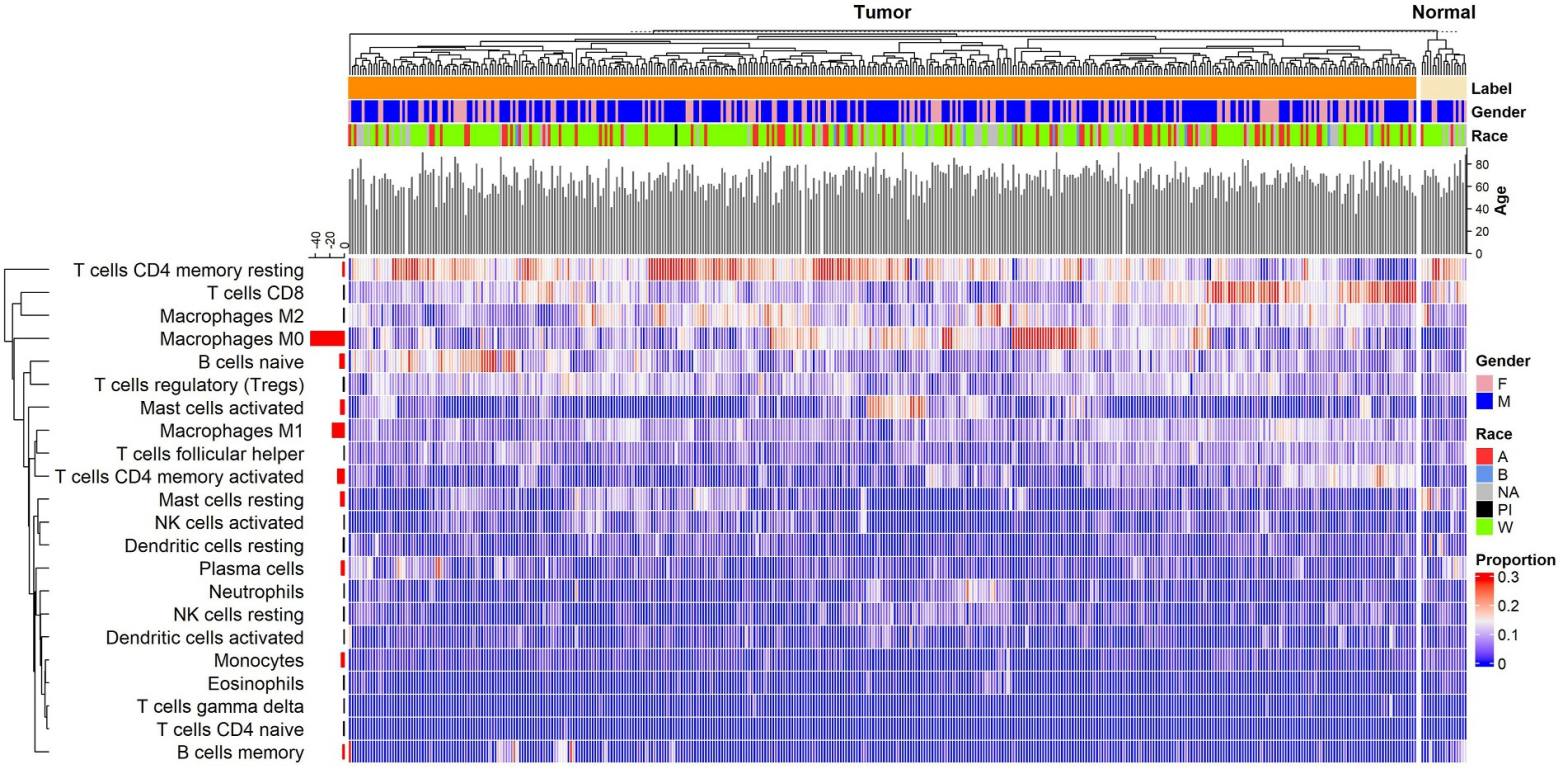
Heatmap of immune cell composition for stomach adenocarcinoma at individual sample resolution. The bar plot on the left denotes the −log_10_‐transformed *t*‐test *p*‐value between tumor and normal samples. Red color indicates significant results, and black color indicates insignificant results. The race codes are as follows: A, Asian; B, Black; NA, not available; PI, Pacific Islander; W, White.

**FIGURE 4:**
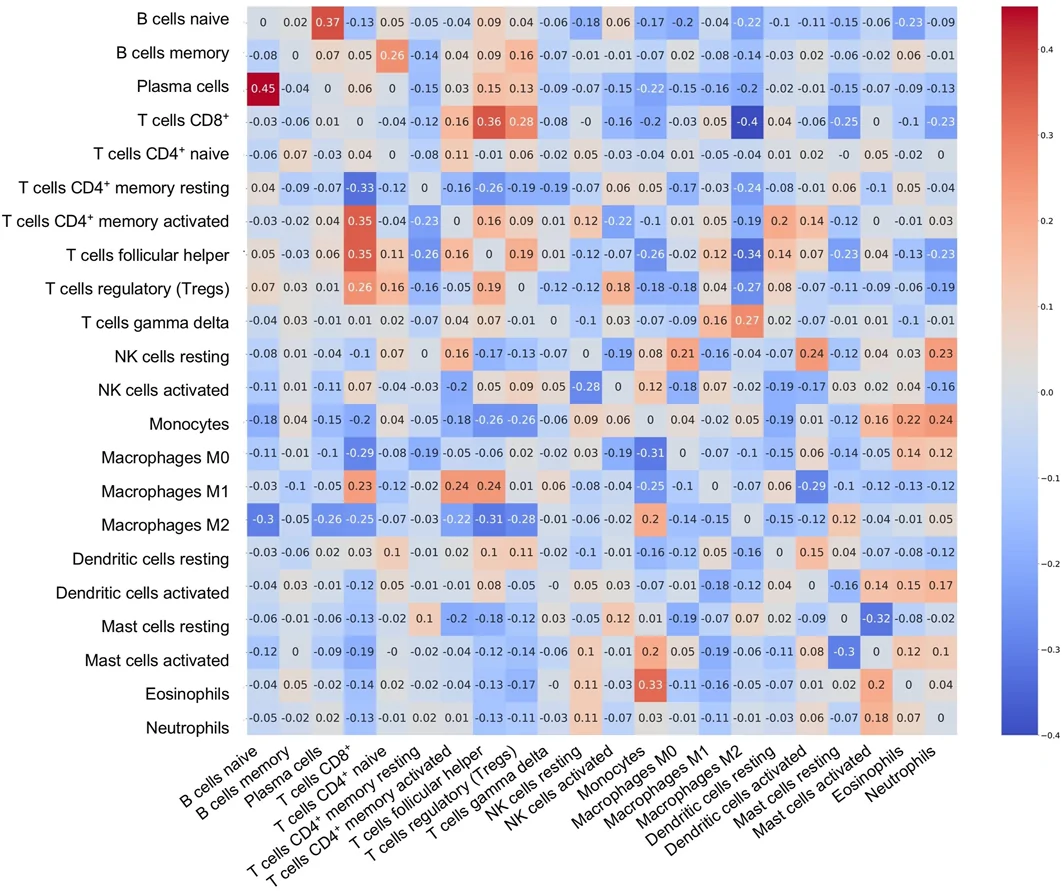
Pairwise correlation matrix between immune cell types for normal (upper right) and tumor (lower left) samples. The correlation was computed by combining all samples from all cancer types.

### 2.2 Immune cell type tumor specificity

During tumorigenesis, significant changes occur in the composition and function of immune cells within the tissue. We measured the differences between tumor and normal samples across 15 cancer types with normal sample sizes greater than or equal to 10. Out of 352 cancer‐cell type combinations, 183 showed significant differences between tumor and normal samples (Figure 5A, Table S3). Further analysis revealed that although most cancer types exhibited consistent directional changes from normal to tumor, some exceptions were observed (Figure 5B). For instance, macrophage M0 cell content increased in all cancer types except lung adenocarcinoma (LUAD) (Figure 5C–H); B cells naive decreased in 11 cancer types but increased in LUAD, lung squamous cell carcinoma (LUSC), and stomach adenocarcinoma (STAD). ResNet [17] models demonstrated that the differences in immune cell composition were strong enough to distinguish between tumor and normal samples (Figure 5I). In these binary models, samples from one specific cancer type are labeled as positive, whereas samples from all other cancer types are labeled as negative. Because of the highly unbalanced ratio of positive to negative samples, accuracy, precision, and recall were not considered suitable metrics. Instead, the more balanced metric, the F1 score, was used to evaluate model performance.

**FIGURE 5:**
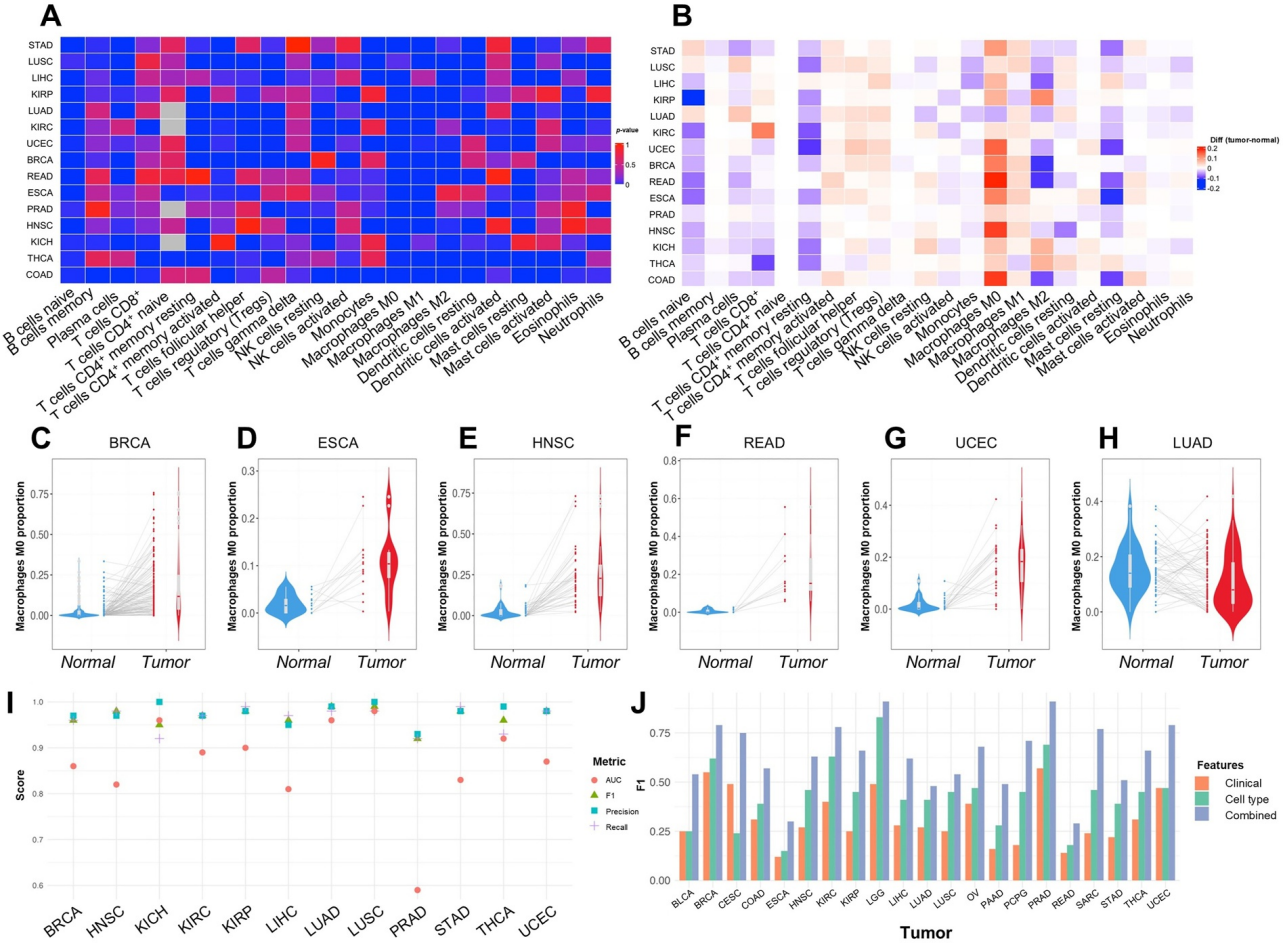
Tumor–normal differences in immune cell composition, M0 macrophage alterations, and ResNet model performance for tumor and cancer type classification. (A) Heatmap of *p*‐values for tumor versus normal immune cell composition. (B) Heatmap of the difference between mean immune cell composition between tumor and normal. (C–G) Violin plots showing changes in macrophage M0 proportions in paired normal–tumor samples. These five cancer types showed increased macrophage M0 proportions in tumors. (H) Violin plot showing changes of macrophage M0 proportions in paired normal–tumor samples in LUAD. In LUAD, macrophage M0 decreased in normal. (I) F1 scores of ResNet models distinguishing tumor and normal samples using immune cell composition. (J) F1 scores of ResNet binary models distinguishing cancer type. LUAD, lung adenocarcinoma.

Cancer is a highly heterogeneous disease, with each type exhibiting subtly different patterns of immune cell infiltration. In this analysis, we examined 21 cancer types, each with a tumor sample size > 150. Our model indicates that although these immune cell patterns are not distinct enough to fully differentiate each cancer type in a multioutcome model, they do contain sufficient cancer‐specific information to construct effective binary outcome models for each cancer type. For the binary models, three types of features were used: (1) clinical variables (age, stage, and race, Figure S1A); (2) immune cell abundance (Figure S1B); and (3) a combination of clinical variables and immune cell abundance (Figure S1C). Because of sex‐specific cancer types (e.g., breast cancer), sex was excluded as a variable. The binary models using only clinical variables achieved an average F1 score of 0.31 (min: 0.12, max: 0.57) (Figure 5J). The models using immune cell types alone performed slightly better, achieving an average F1 score of 0.43 (min: 0.15, max: 0.83). Substantial performance improvement was obtained by combining clinical and immune cell type features, resulting in an average F1 score of 0.64 (min: 0.29, max: 0.91). We also conducted logistic regression analyses to separate cancer type. The resulting F1 scores averaged 0.26 (min: 0.05, max: 0.84) and were worse than the ResNet results.

### 2.3 Immune cell prognosis

Cox proportional hazards analyses for disease‐specific survival (DSS) were conducted for each cancer type and cell type combination, adjusting for age, sex, race, and stage. Cancer types with sample sizes < 100 were excluded from the analysis. A Benjamini–Hochberg [18] adjusted *p*‐value of < 0.05 was considered statistically significant. After removing statistically unstable results (hazard ratio [HR] near 0 or unrealistically large), significant associations were identified (Figure 6). All four results showed that a lower immune cell proportion corresponded to a better prognosis. In bladder urothelial carcinoma (BLCA), macrophage M0 (adjusted *p* = 0.03, HR [95% CI] = 12.33 [2.87–52.93]) (Figure 6A) and macrophage M2 (adjusted *p* = 0.04, HR [95% CI] = 44.5 [4.66–425.36]) (Figure 6B) were significantly associated with DSS. In kidney renal clear cell carcinoma (KIRC), macrophage M0 was significantly associated with DSS (adjusted *p* = 0.02, HR [95% CI] = 28.29 [24.55–175.96]) (Figure 6C). In skin cutaneous melanoma (SKCM), the Treg/CD8^+^ T cell ratio was significantly associated with DSS M0 (adjusted *p* = 0.01, HR [95% CI] = 25.69 [4.55–141.96]) (Figure 6D). The Treg/CD8^+^ T cell ratio is a widely studied immunological marker in cancer prognosis, reflecting the balance between immune suppression and antitumor immunity within the TME. Tregs are known for their immunosuppressive functions, which help maintain immune tolerance but can also inhibit effective antitumor responses. In contrast, CD8^+^ T cells are cytotoxic lymphocytes that directly kill tumor cells. A high Treg/CD8^+^ T cell ratio suggests a microenvironment dominated by immune suppression, often correlating with poor prognosis, tumor progression, and resistance to immunotherapy [19].

**FIGURE 6:**
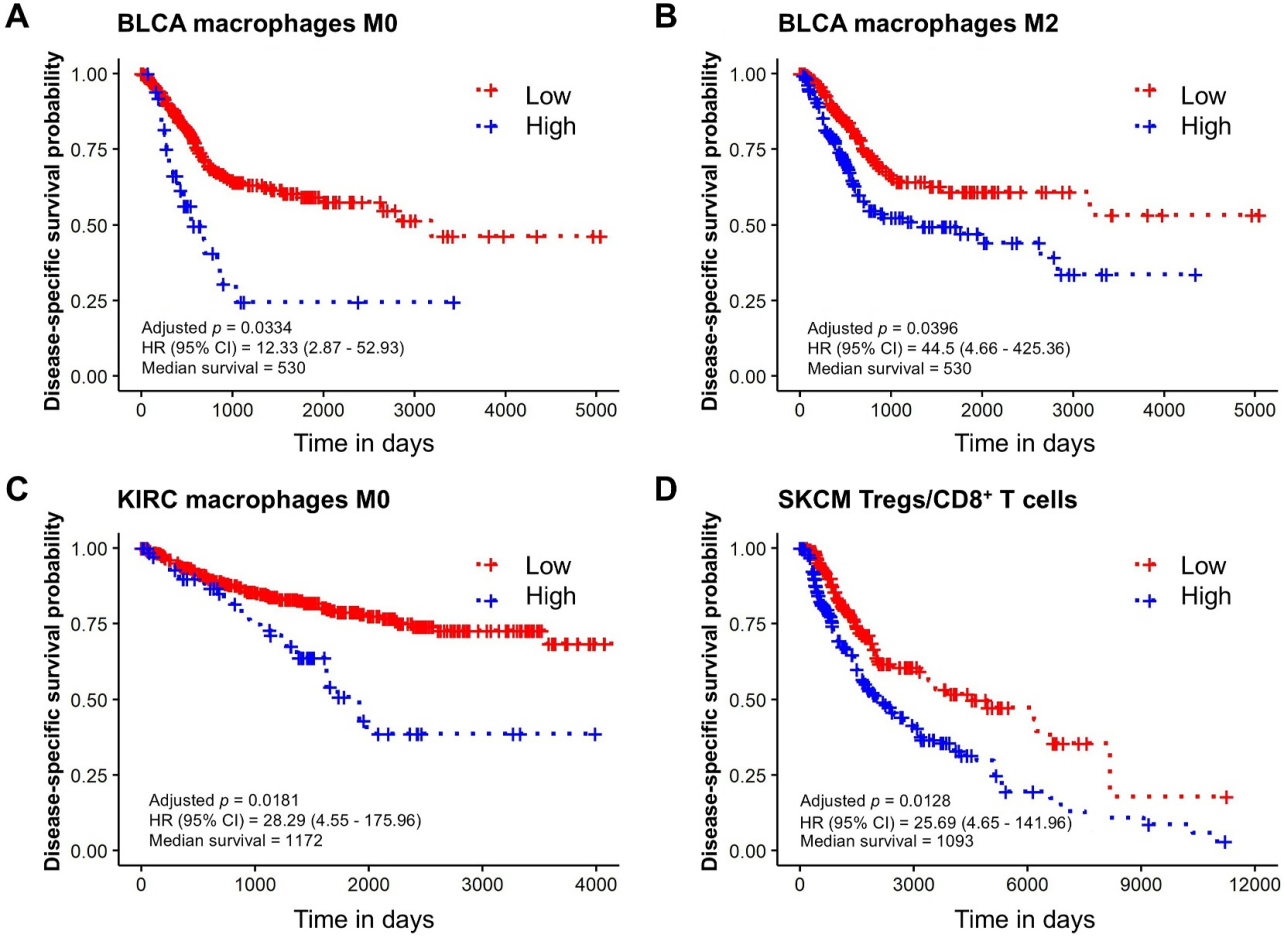
Kaplan–Meier curves of four immune features significantly associated with disease‐specific survival in Cox proportional hazards analysis. *p*‐values are from Cox regression. (A) Macrophage M0 in BLCA. (B) Macrophage M2 in BLCA. (C) Macrophage M0 in KIRC. (D) Treg/CD8^+^ T cell ratio in SKCM. BLCA, bladder urothelial carcinoma; KIRC, kidney renal clear cell carcinoma; SKCM, skin cutaneous melanoma.

Macrophage M0 is considered undifferentiated or naive, representing a transitional state before polarization into either proinflammatory (M1) or anti‐inflammatory (M2) phenotypes. A high abundance of M0 macrophages in the TME may reflect a dysfunctional immune response, where macrophages fail to polarize effectively and instead contribute to a permissive environment for tumor growth. This could explain the observed HR of 12.33, indicating that patients with elevated M0 macrophage levels are over 12 times more likely to experience disease‐specific mortality compared to those with lower levels. Macrophage M2, on the other hand, is well established as immunosuppressive and protumorigenic. They facilitate tumor progression by promoting angiogenesis, tissue remodeling, and suppression of cytotoxic T cell activity. The HR of 44.5 for M2 macrophages is particularly striking, suggesting a dramatically increased risk of disease‐specific death in patients with high M2 infiltration. Although the confidence interval is wide, reflecting variability and possibly limited sample size, the association remains statistically significant and biologically plausible.

### 2.4 Immune cell diversity

Immune cell diversity may influence cancer behavior. We measured immune cell diversity using the Shannon index for all cancer types (Figure 7A), finding an average Shannon index of 2.10. LUAD exhibited the highest diversity with a Shannon index of 2.28, whereas brain lower grade glioma (LGG) had the lowest at 1.73. Pairwise comparisons of immune cell diversity between cancer types were conducted, revealing that 344 of the 528 possible cancer pairs from the 33 cancer types showed significant differences (Figure 7B). Immune cell diversity differences between tumor and normal tissue were also assessed within each cancer type, with significant differences identified in five cancer types (Figure 7C–G). In thyroid carcinoma (THCA), prostate adenocarcinoma (PRAD), BLCA, and breast invasive carcinoma (BRCA), tumors had a higher Shannon index than normal tissue, whereas in kidney renal papillary cell carcinoma (KIRP), normal tissue had a higher Shannon index than tumors. DSS analyses were conducted with the Shannon index. No statistically significant results were identified.

**FIGURE 7:**
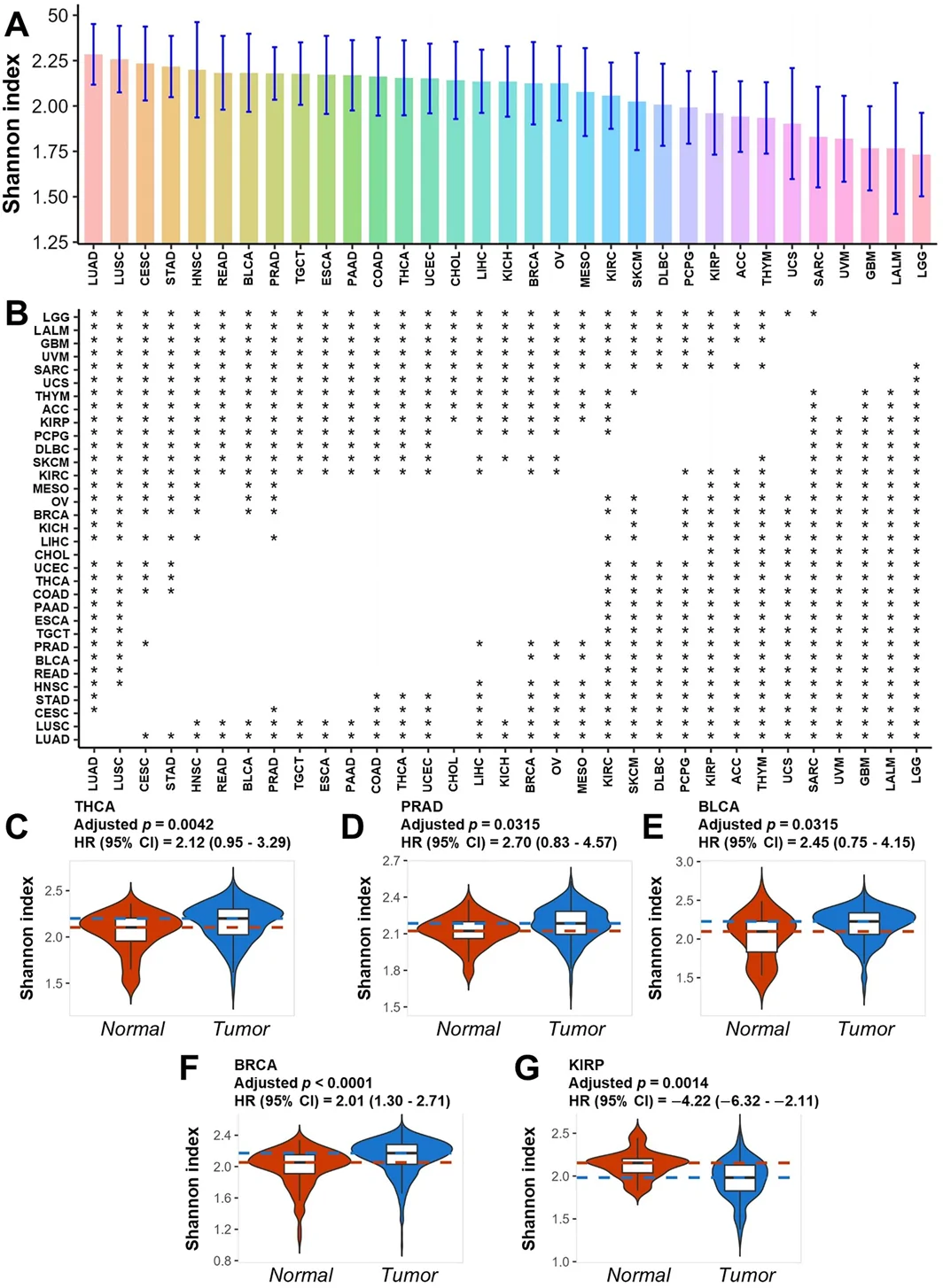
Immune cell diversity across cancer types measured by the Shannon index, highlighting cancer pairs and tumor–normal differences in significant cases. (A) Immune cell diversity was measured as the Shannon index. The mean Shannon index in each cancer type is displayed as a bar. (B) Cancer pairs showed significant immune cell diversity differences. (C–G) Violin plots that display the five significant results of the Shannon index difference between tumor and normal samples.

## 3 DISCUSSION

Cancer heterogeneity refers to the existence of diverse characteristics within and between tumors in cancer patients. This diversity can manifest in various aspects, such as genetic, epigenetic, phenotypic, and environmental differences. Understanding cancer heterogeneity is crucial for diagnosing, treating, and managing cancer, as it impacts how tumors respond to therapies and their overall behavior. Cancer heterogeneity has been widely studied in genomics, transcriptomics, epigenomics, and proteomics. In this study, we aim to describe cancer heterogeneity through immune cell composition inferred through RNA‐seq deconvolution. These cells interact with tumor cells in complex ways, influencing tumor growth, progression, and response to treatment. When normal tissue transforms into a tumor, significant changes occur in the composition and function of immune cells within the tissue. These changes are influenced by the tumor’s ability to evade the immune system, promote inflammation, and create an immunosuppressive microenvironment.

Recent research has underscored the complex relationship between immune cell composition, diversity, and cancer prognosis. The Shannon index, a widely used measure of immune diversity, captures both the richness and evenness of immune cell populations within the TME. Our pan‐cancer analysis revealed that immune cell diversity varies significantly across cancer types: Some tumors display highly diverse immune landscapes, whereas others are dominated by a few cell types. Importantly, the prognostic value of immune diversity is context‐dependent; higher diversity correlates with improved survival in certain cancers but not in others. This variability likely reflects the multifaceted mechanisms of tumor immune escape. Tumors with low immune diversity are often dominated by immunosuppressive cell types, such as regulatory T cells or M2 macrophages, which promote immune evasion and resistance to therapy. In contrast, more diverse infiltrates, enriched for CTLs and antigen‐presenting cells, are generally indicative of stronger antitumor immunity and improved clinical outcomes. Notably, immune diversity has been associated with response to immune checkpoint inhibitors (ICIs), with studies showing that tumors enriched in multiple immune cell types, particularly T cells, are more likely to benefit from anti‐PD‐1/PD‐L1 or anti‐CTLA‐4 therapies [10, 11].

In bladder cancer (BLCA), our Cox proportional hazards analysis provided further mechanistic insight into how specific immune populations shape prognosis. We identified macrophage subtypes M0 and M2 as being strongly associated with DSS. After adjusting for clinical covariates and correcting for multiple testing, higher proportions of these macrophages were significantly linked to poorer survival outcomes, with HRs of 12.33 for M0 and 44.5 for M2 macrophages. These findings align with and extend previous reports on tumor‐associated macrophages (TAMs) as key mediators of tumor progression. TAMs, among the most abundant infiltrating immune cells in solid tumors, exhibit polarization states ranging from proinflammatory M1‐like to immunosuppressive M2‐like phenotypes. M2‐like TAMs, in particular, have been implicated in promoting tumor growth, angiogenesis, and immune evasion across cancers [20]. In BLCA specifically, Leblond et al. [20] demonstrated that M2 macrophages secrete anti‐inflammatory cytokines and immune inhibitory molecules such as PD‐L1, thereby suppressing cytotoxic T cell activity and facilitating tumor escape. This mechanistic evidence supports our observation that elevated M2 macrophage infiltration contributes to poor prognosis, highlighting how both immune diversity metrics and specific immunosuppressive subpopulations serve as critical determinants of patient outcomes.

Across the 15 cancer types with adequate normal controls, M0 macrophages were elevated in tumors compared with matched normal tissues in nearly all cancers except LUAD, where M0 proportions decreased, highlighting a tissue‐context–dependent trajectory of macrophage differentiation in the TME. This pattern suggests that LUAD harbors microenvironmental cues that accelerate macrophage polarization away from an undifferentiated M0 state toward functionally committed phenotypes, thereby depleting the detectable M0 pool in bulk deconvolution [21]. Unlike many solid organs, the lung hosts a large pool of alveolar macrophages (AMs), long‐lived, tissue‐resident macrophages that are developmentally and functionally distinct from monocyte‐derived TAMs [22]. AM identity and homeostasis depend on epithelial granulocyte–macrophage colony‐stimulating factor (encoded by the *CSF2* gene) and PPARγ‐driven lipid metabolic programs that are unique to the surfactant‐rich alveolar niche, and these cues are maintained or co‐opted in LUAD to rapidly imprint suppressive phenotypes [23]. In practical terms, such strong niche signals likely push infiltrating or newly recruited macrophages through an abbreviated “M0” window into M2‐like immunoregulatory states (e.g., IL‐10–high, PD‐L1–high), which would present in bulk deconvolution as lower M0 and higher polarized subsets, consistent with our LUAD observation.

The LUAD milieu is enriched for metabolic features (hypoxia, lactate, lipids) that are known to reprogram macrophage fate via HIF‐1α– and NF‐κB–coupled pathways, favoring M2‐like polarization and functional immunosuppression [24]. Tumor‐derived lactate further drives macrophages toward an anti‐inflammatory program and dampens toll‐like receptor/NF‐κB signaling, again minimizing residence time in an M0‐like state [25, 26]. These metabolic constraints are more pronounced in the lung’s ventilated, surfactant‐rich niche than in several other tissues, providing a plausible explanation for why LUAD diverges from the pan‐cancer trend.

LUAD subtypes show distinct stromal programs that influence macrophage recruitment and fate. For instance, *STK11*/*LKB1* loss in *KRAS*‐mutant LUAD produces a cold TME with neutrophil accumulation and T cell suppression, conditions under which monocyte‐to‐macrophage differentiation can be diverted and rapidly polarized, thereby underrepresenting an M0‐like intermediate [27, 28]. Conversely, *EGFR*‐mutant LUADs are characterized by TAM enrichment and cytokine axes (e.g., CSF1/CSF1R, CCL2/CCR2) that promote M2 skewing and resistance to EGFR‐TKIs, consistent with swift commitment beyond an M0 state [29]. Single‐cell and preclinical work further indicates that *EGFR*‐driven tumors co‐opt AMs, reinforcing tissue‐resident programs that are not well captured by peripheral blood–derived reference matrices [30].

Macrophage polarization is tightly regulated by danger signals and cytokines such as TNFα, which exerts pleiotropic, context‐dependent effects via MAPK/Akt/NF‐κB signaling; high and sustained TNFα can sustain M1‐like features, whereas distinct TNFα dynamics or cross‐talk with IL‐10/TGF‐β can bias toward immunoregulatory states [31]. In LUAD, chronic epithelial injury, airway exposures, and tumor‐derived IL‐10/TGF‐β likely attenuate proinflammatory TNFα outputs or redirect downstream signaling, facilitating more rapid departure from an M0 state than in other tissues. The miRNA landscape, shaped by tumor genetics, environmental exposure, and stromal cross‐talk, modulates macrophage polarization networks (e.g., NF‐κB, STATs, PPARs) and may explain variable M0 prevalence across cancers [32]. For example, miRNAs controlled by bioactive small molecules (e.g., resveratrol) highlight how post‐transcriptional circuits can push macrophages toward defined states; although not lung‐specific, these principles support a model where LUAD‐specific miRNA milieus hasten polarization beyond M0 [32].

Mechanistic reviews of curcumin show that targeted modulation of MAPK/NF‐κB signaling can repolarize macrophages and shift the M1/M2 balance, reinforcing the idea that pathway context, not just cell lineage, governs macrophage state distributions at steady state and under tumor pressure [33]. Extrapolating to LUAD, strong endogenous modulators in the airway (e.g., lipids, oxidants, smoke‐related irritants) likely act as “physiologic pathway modulators,” compressing the M0 window and manifesting as lower M0 abundance in deconvolution. Our deconvolution relied on LM22, which was derived from peripheral blood immune populations and does not explicitly model AMs or other lung TRMs, raising the possibility that AM‐like signatures are projected onto polarized bins (e.g., M2) rather than M0. This limitation would accentuate the LUAD exception if AMs constitute a large share of intratumoral macrophages as suggested in lung cancer while having minimal representation in other tissues.

Our study contributes several novel insights that extend the current literature. First, by analyzing 33 cancer types and over 11,000 samples, we present one of the most comprehensive pan‐cancer immune deconvolution efforts to date. Although previous studies such as those by Thorsson et al. [11] and Gentles et al. [8] also utilized TCGA data, our work uniquely integrates immune cell composition, immune diversity (via the Shannon index), and deep learning‐based classification into a unified analytical framework. Second, we employed a 1D ResNet deep learning model optimized through Bayesian search to classify tumor versus normal tissue and to distinguish cancer types in binary classification tasks. This approach demonstrates that immune cell composition, especially when combined with clinical features, can meaningfully differentiate tissue states and cancer types, an insight not deeply explored in prior studies. Third, our use of the Shannon index to quantify immune cell diversity and its association with survival outcomes (e.g., in KIRP) introduces a novel dimension to immune profiling. Although earlier studies focused on specific immune cell types or gene signatures, our findings suggest that overall immune diversity itself may serve as a biologically and clinically relevant prognostic feature. Fourth, our analysis of tumor versus normal immune cell composition across 15 cancer types reveals both consistent and divergent remodeling patterns. For instance, macrophage M0 levels increased in most cancers but decreased in LUAD, highlighting the context‐dependent nature of immune responses. This nuanced view of immune remodeling adds depth to the field’s understanding of tumor immunology. Fifth, the identification of 16 significant immune cell–survival associations across multiple cancer types provides a more comprehensive and comparative perspective than most prior studies, which often focus on a single cancer or immune subset. These associations span both protective and detrimental roles for macrophages, T cells, mast cells, and B cells, reinforcing the complexity of immune–tumor interactions. Finally, our conclusion that no unifying immune pattern exists across all cancers reinforces the need for cancer type‐specific immune profiling. This finding aligns with the immune subtype framework proposed by Thorsson et al. [11], but our results further emphasize the limitations of pan‐cancer generalizations and the importance of tailored immunological assessments.

Our study also contributes to the novel biomarker research for cancer therapy. The integration of multiomics and network pharmacology is transforming cancer research, enabling the discovery of novel biomarkers and therapeutic targets. Our study, using transcriptome deconvolution and deep learning across 33 cancer types, underscores the prognostic value of immune cell composition and its potential for guiding therapy. Recent advances in multiomics combining genomics, transcriptomics, proteomics, and metabolomics have proven essential for identifying disease subtypes and actionable biomarkers, especially through single‐cell and spatial analyses that reveal cell type‐specific features not seen in bulk data [34]. Our identification of macrophage M0/M2 subtypes and the Treg/CD8^+^ T cell ratio as prognostic markers fits this paradigm, highlighting immune features that can inform patient stratification and immunotherapy. Our findings have direct translational relevance as immune landscape assessments can help select patients most likely to benefit from ICIs or combination therapies and guide the development of tailored treatment strategies. As Li et al. [35] emphasize, the immune microenvironment is a dynamic and actionable target for intervention, and its characterization is essential for advancing precision oncology.

Network pharmacology further enhances target discovery by mapping complex interactions among genes, proteins, and metabolites, moving beyond the traditional “one drug‐one target” model [34]. Integrating omics with network analysis has revealed new metabolic and protein targets, as well as actionable gene signatures, across multiple cancer types. Targeting immune cell subtypes or their regulatory pathways such as M2 macrophage polarization or the Treg/CD8^+^ T cell ratio balance may offer new therapeutic avenues. The growing use of AI and machine learning with multiomics and network pharmacology is accelerating biomarker discovery and the development of personalized cancer therapies.

Despite the strengths of our study, several limitations should be noted. First, the use of the LM22 reference matrix in CIBERSORTx restricts immune profiling to only 22 predefined immune cell types, omitting rare or tissue‐specific populations such as myeloid‐derived suppressor cells or exhausted T cells, which may play critical roles in cancer progression. Additionally, LM22 was derived from peripheral blood immune cells and may not fully capture the transcriptional diversity of tumor‐infiltrating immune cells in solid tumors. CIBERSORTx also provides only relative estimates, with immune cell fractions summing to one per sample, which can obscure absolute abundance differences. Moreover, any cell types not included in the reference matrix are assumed to be absent, potentially overlooking important components of the TME. Another limitation is the lack of validation in an independent dataset due to the scarcity of large RNA‐seq cohorts with complete survival data. Nonetheless, our findings offer a robust and interpretable framework for understanding immune heterogeneity in cancer and its implications for prognosis and therapy.

## 4 CONCLUSION

Our study presents a comprehensive analysis of immune cell composition across 33 cancer types using RNA‐seq deconvolution, revealing significant differences between tumor and normal tissues and identifying 16 immune cell–survival associations. By integrating immune diversity metrics and deep learning classification, we demonstrate that immune profiles can meaningfully distinguish tissue states and inform prognosis. Our findings highlight the biological relevance of immune cell diversity, the context‐dependent roles of specific immune subsets, and the absence of a universal immune signature across cancers, emphasizing the need for cancer type‐specific immune profiling to guide personalized therapeutic strategies.

## 5 MATERIALS AND METHODS

### 5.1 Data collection and preprocessing

Transcript per million normalized RNA‐seq transcriptomic data for 11,274 patients spanning 33 cancer types (ACC, BLCA, BRCA, CESC, CHOL, COAD, DLBC, ESCA, GBM, HNSC, KICH, KIRC, KIRP, LAML, LGG, LIHC, LUAD, LUSC, MESO, OV, PAAD, PCPG, PRAD, READ, SARC, SKCM, STAD, TGCT, THCA, THYM, UCEC, UCS, UVM) were obtained from the TCGA program in the Genomic Data Commons portal. Expression profiles included quantification for 59,427 genes per sample. Immune cell composition was inferred using CIBERSORTx [16] with the LM22 signature matrix provided by CIBERSORTx, which estimates the relative fractions of 22 immune cell types from bulk tissue RNA‐seq data. These estimates were normalized such that the proportions summed to one per sample. To quantify overall immune cell diversity, we computed the Shannon diversity index for each sample. To assess immune–tumor dynamics, we performed differential abundance analysis using two‐sided *t*‐tests to compare immune cell proportions between tumor and matched normal tissues. For survival analysis, Cox proportional hazards regression was applied to assess associations between immune cell proportions and DSS, adjusting for age, race, sex, and tumor stage. When presenting survival analysis results, Kaplan–Meier curves were used. The dichotomization of the data in Kaplan–Meier analysis was performed using the R function *surv_cutpoint* from the survminer package. The *p*‐values and HRs reported in Kaplan–Meier curves were from Cox regression. The statistical significance threshold was a Benjamini–Hochberg [18] adjusted *p*‐value < 0.05.

For machine learning tasks, the CIBERSORTx‐derived immune cell fractions were used as features in two classification settings: cancer type classification (distinguishing among primary tumor types) and tumor versus normal classification within individual cancer types. For cancer type classification, we selected 21 cancer types with > 150 tumor samples to ensure sufficient representation and model stability. For tumor versus normal classification, we selected 13 cancer types that each had ≥ 20 normal tissue samples. Three types of input features were tested: (i) clinical variables, (ii) immune cell proportions, and (iii) a combination of both. Clinical variables included age at diagnosis (15–90), race (Asian, Black or African American, White, Native Hawaiian or other Pacific Islander, and not available), and tumor stage (TNM classification). Categorical variables were one‐hot encoded, and missing values were imputed (mean imputation for age; “Unknown” category for race and stage). Sex was excluded to avoid confounding from sex‐specific cancers (e.g., breast, prostate). Data were partitioned into training and test sets using an 80/20 split, stratified by class labels to preserve group proportions. To address class imbalance in the training data (particularly in tumor vs. normal classification), we applied the synthetic minority oversampling technique [36] to generate synthetic instances for underrepresented classes.

### 5.2 Model architecture and optimization

We employed a custom 1D residual neural network (ResNet) [17] architecture tailored for each binary classification task. The architecture was designed for flexibility, interpretability, and performance and was optimized for each task using Bayesian optimization. For cancer type classification, one multiclass dataset was used, whereas for tumor versus normal classification, 13 separate binary datasets (one per cancer type) were constructed. Each model began with an input layer that matched the dimensionality of the feature space, followed by 1–3 hidden layers containing 8–64 neurons, as determined through optimization. Hidden layers included the following: batch normalization to accelerate convergence and stabilize training; ReLU activation for nonlinearity and to mitigate vanishing gradient issues; dropout (0.0–0.5 range) to reduce overfitting; and residual (skip) connections between selected layers to improve gradient flow and model depth tolerance. The final layer consisted of a sigmoid‐activated output node, producing a probability score suitable for binary classification. The model was compiled with the Adam optimizer, and learning rates were tuned across (0.01, 0.001, 0.0001). Binary cross‐entropy was used as the loss function. Each model was trained for up to 200 epochs with early stopping triggered by a lack of validation loss improvement for 20 consecutive epochs.

### 5.3 Optimization and model evaluation

Hyperparameters and architecture configurations were optimized using the Bayesian tuner from KerasTuner, which balances exploration and exploitation of the hyperparameter space. For each task, multiple candidate models were evaluated using cross‐validation on the training set, with selection based on the F1 score, which balances precision and recall and is robust to class imbalance. Final model evaluation was performed on the held‐out test set. For each task, we selected the top five models identified by the tuner and reported the average F1 score to provide a more stable performance estimate. All model training and evaluation were performed using the TensorFlow and Keras frameworks. This approach ensured robust and reproducible model selection across cancer types and classification tasks.

### 5.4 Candidate architecture evaluation

Candidate architectures were evaluated based on their performance on the hold‐out test set, using the F1 score as the primary metric. For cancer type classification, we used a one‐vs‐rest strategy to accommodate multiclass evaluation. The best models identified by the Bayesian tuner were further assessed on test data to confirm generalizability to unseen samples. To account for variability in optimization and initialization, we selected the top five performing models for each task and reported the average F1 score as the final performance measure. This rigorous, multistage evaluation process ensured robust training, validation, and assessment of deep learning models across all classification tasks.

## AUTHOR CONTRIBUTIONS

**Limin Jiang**: Data Curation; investigation; methodology; software; formal analysis; visualization; writing—original draft. **Chung‐I Li**: Formal Analysis; methodology; writing—review and editing. **Rui Xin**: Methodology; investigation; software; validation; formal analysis; visualization **Jijun Tang**: Conceptualization; resources; supervision. **Yan Guo**: Conceptualization; funding acquisition; methodology; project administration; resources; supervision; writing—review and editing.

## CONFLICT OF INTEREST STATEMENT

The authors declare no conflicts of interest.

## ETHICS STATEMENT

No studies involving human participants or animals were performed by any of the authors.

## Supporting information

Figure S1

Table S1

Table S2

Table S3

## Data Availability

All data used in this study were downloaded from Genomic Data Commons.
