# Supplementary material for: Cancer heterogeneity explored through immune cell deconvolution of the tumor microenvironment
Source: Quant Biol. 2026 Apr 15;14(3):e70037. doi: 10.1002/qub2.70037 (PMC13642144; doi:10.1002/qub2.70037)
Supplement: Supplementary file 1 [file QUB2-14-e70037-s003.docx]

**Supplementary Figure S1**. Description of ResNet models with three types of input. (A) clinical variables: age, stage, and race; (B) Immune cell abundance; and (C) a combination of clinical variables and immune cell abundance.
